# Effects of music therapy on anxiety among patients undergoing cardiac procedures: a systematic review and meta-analysis

**DOI:** 10.1097/MS9.0000000000003226

**Published:** 2025-04-02

**Authors:** Zoaib Habib Tharwani, Prince Kumar, Avinash Kumar, Saad Javaid, Rohet Kumar, Mohsin Ali Shah, Sean Kaisser Shaeen, Fnu Sunita, Naresh Kumar Ladhwani, Abdullah Malikzai

**Affiliations:** a Faculty of Medicine, Dow Medical College, Dow University of Health Sciences, Karachi, Pakistan; b Bahria University Medical and Dental College, Karachi, Pakistan; c Lahore Medical and Dental College, Lahore, Pakistan; d Shaheed Mohtarma Benazir Bhutto Medical College Lyari, Karachi, Pakistan; e Cambridge University Hospitals NHS Foundation Trust, Addenbrooke’s Hospital, Cambridge, UK; f Kabul University of Medical Sciences, Kabul, Afghanistan

**Keywords:** anxiety, coronary artery bypass graft, music therapy, percutaneous coronary intervention

## Abstract

**Objective:**

The article aims to assess impact of music therapy on anxiety in patients undergoing cardiac procedures

**Methods:**

MEDLINE, Embase, and Cochrane Central were searched to identify relevant cohorts and randomized controlled trials (RCTs). We evaluated anxiety, heart rate (HR), systolic and diastolic blood pressures (SBP and DBP), respiratory rate, mean arterial pressure (MAP), and mean oxygen saturation (SaO_2_) using weighted random-effects models, reporting mean difference (MD) with 95% confidence intervals.

**Results:**

In total, 1706 patients from 18 RCTs and one cohort study were analyzed. Music therapy recipients showed significantly lower anxiety measured by the State-Trait Anxiety Inventory (MD: −4.75; *P* = 0.002). The music therapy group demonstrated improved HR (MD: −4.90, *P* = 0.02), SBP (MD: −5.14, *P* = 0.01), and respiratory rate (MD: −1.15, *P* = 0.01). No significant differences were observed in anxiety measured by the Numerical Anxiety Rating Scale (MD: −1.09, *P* = 0.14), DBP (MD: −2.61, *P* = 0.15), MAP (MD: −4.71, *P* = 0.47), or SaO_2_ (MD: 0.93, *P* = 0.61).

**Conclusion:**

Music therapy significantly reduces anxiety and enhances post-procedural HR, SBP, and respiratory rates in cardiac procedure patients.

## Introduction

In modern world, cardiovascular disorders are the primary cause of mortality^[1]^. One of the most beneficial and dependable cardio-invasive examinations is cardiac catheterization, which offers crucial details regarding ventricular function, congenital disorders, valvular heart illnesses, and coronary artery disease^[2]^. Many individuals experience anxiety when hospitalized^[3]^. As patients anticipate their cardiac catheterization, anxiety levels increase during the waiting period^[4]^. Since the patient is cognizant, sounds from the surgical instruments and other team members’ interactions may unintentionally create a stressful situation for them^[2]^. Patients’ past experiences, discomfort, stress, unusual surroundings, dread of the unknown, outcome of treatments, the necessity of surgery, worry about complications, and an uncertain future are the most frequent causes of anxiety in patients^[5]^.

**HIGHLIGHTS**

- Music therapy reduces anxiety during cardiac procedures, showing significant benefits.
- Meta-analysis confirms marked reduction in State-Trait Anxiety Inventory scores with music therapy.
- Integration of music therapy in clinical settings is recommended for anxiety management.

Anxiety during a crucial procedure raises the possibility of both physical and psychological harm^[6]^. Anxiety is a mood condition marked by feelings of tension, irritation, stress, and fear, as well as elevated norepinephrine and adrenaline plasma levels and heightened autonomic nervous system activity^[7]^. Severe anxiety warrants sedative use both before and during the procedure^[8]^. Anxiety increases pulse, blood pressure, and cardiac output and aggravates the burden on the heart^[9]^.

These days, more people are willing to adopt non-pharmacological anxiety relief techniques. Using music therapy, a desirable sound stimulant, is one of these techniques^[10]^. As a suitable and successful intervention, music is introduced and utilized in several departments, particularly in general and psychiatric hospitals and rehabilitation centers, where it helps with physical, psychological, and cognitive diseases^[11]^. Patients experiencing problems like stress, irritation, loneliness, mood improvement, and enthusiasm facilitation might benefit from music therapy. By raising the threshold for stress, controlling internal processes, inducing relaxation, and enhancing the release of different hormones, such as endorphins, supplementary therapy enhances patient comfort and safety^[12]^. Music fortifies the immune system and all of its operations as well. Using non-pharmacological techniques like music therapy and other techniques can also help lower blood pressure and the dosage of medication required^[13]^.

Anxiety can lead to undesirable outcomes during cardiac-invasive procedures, and addressing this issue is imperative to improve patient outcomes. In this systematic review and meta-analysis, we investigate the impact of music therapy on anxiety levels in patients undergoing cardiac procedures, such as percutaneous coronary intervention (PCI) and coronary artery bypass graft (CABG), aiming to provide a comprehensive synthesis of existing research to guide evidence-based interventions and enhance the overall well-being of individuals in this medical context. We also consider other outcomes, including the effects of music therapy on heart rate (HR), blood pressure, and respiratory rate (RR) among the same population.

## Methods

The Preferred Reporting Items for Systematic Review and Meta-Analyses (PRISMA) guidelines and the Risk of Bias in Systematic Reviews and Assessment of Multiple Systematic Reviews (AMSTAR) 2 were both followed while doing this meta-analysis^[14,15]^.

### Data sources and search strategy

MEDLINE, Embase, and Cochrane Central were comprehensively searched from inception through October 2023 by two independent reviewers (M.A.S. and Z.H.T.). We extracted studies based on abstracts and titles. A full-text appraisal was sought when required. For a literature search, the following search string was used: (“anxiety” OR “fear” OR “psychological distress” OR “emotional stress” OR “pain” OR “discomfort”) AND (“cardiac surgical procedures” OR “cardiac surgery” OR “coronary artery bypass” OR “coronary artery bypass grafting” OR “open heart surgery” OR “percutaneous coronary intervention”) AND (“music” OR “music therapy” OR “complementary therapies”). To avoid missing any articles, we also searched the references of the included studies.

### Study selection

#### Data extraction and assessment of study quality

We included studies if they were (1) randomized controlled trials (RCTs), non-RCTs, and cohort or analysis that determined the safety and efficacy of music interventions versus no music or conventional care for in different interventional arms, (2) reported either of anxiety levels using the State-Trait Anxiety Inventory (STAI), anxiety using the Numerical Anxiety Rating Scale (NRS-A), HR, systolic blood pressure (SBP), diastolic blood pressure (DBP), RRs, mean arterial pressure (MAP), and mean oxygen saturation (SaO_2_), (3) included patients undergoing cardiac surgery or PCIs, and (4) articles with English titles and abstracts. We excluded all review articles, case reports, case series, editorials, commentaries, and animal-based studies. A third investigator (P.K.) was consulted in case of any disagreement regarding study selection. All articles were then uploaded to Endnote Reference Library (Version X7.5; Clarivate Analytics, Philadelphia, Pennsylvania) software to remove duplicates.

Two reviewers (M.A.S. and Z.H.T.) independently extracted the characteristics of the studies from the selected studies, including patient demographics, summary events, number of events, sample sizes, and treatment types. Summary events were also extracted for outcomes of interest and mean difference (MD) with standard deviation (SD) from the baseline. The quality assessment of the included studies was conducted through the Newcastle-Ottawa scale for cohort 13, the Cochrane risk of bias tool for RCT (Table S1 and Fig. S1, http://links.lww.com/MS9/A783)^[16]^. None of the studies provided follow-up details.

### Statistical analysis

RevMan (version 5.4.1; Copenhagen: The Nordic Cochrane Centre, The Cochrane Collaboration) was used for the meta-analysis. The outcomes of interest were provided as risk ratios with 95% confidence intervals (CIs) and were aggregated using an inverse variance-weighted random-effects model. Forest plots were used to graphically display the pooled analyses. Continuous outcomes of interest were presented as MDs with 95% CIs and were pooled using an inverse variance-weighted random-effects model. When the mean was not available, we used the median for analysis. When the change from the baseline was not reported, we calculated the difference in means between the baseline and the post-treatment measurements. Its SD was derived from the baseline and the follow-up by assuming their correlations were 0.5. Sensitivity and subgroup analyses were performed with different controls where heterogeneity was present. The Higgins *I*^2^ was utilized to assess heterogeneity between trials, and funnel plots were used to assess publication bias for the outcome of anxiety assessed by STAI (Fig. S2, http://links.lww.com/MS9/A783). A 25–50% number was considered low, 50–75% moderate, and >75% serious. In all cases, a *P*-value of less than 0.05 was considered significant.

## Results

### Search results and baseline characteristics

**Figure 1.**
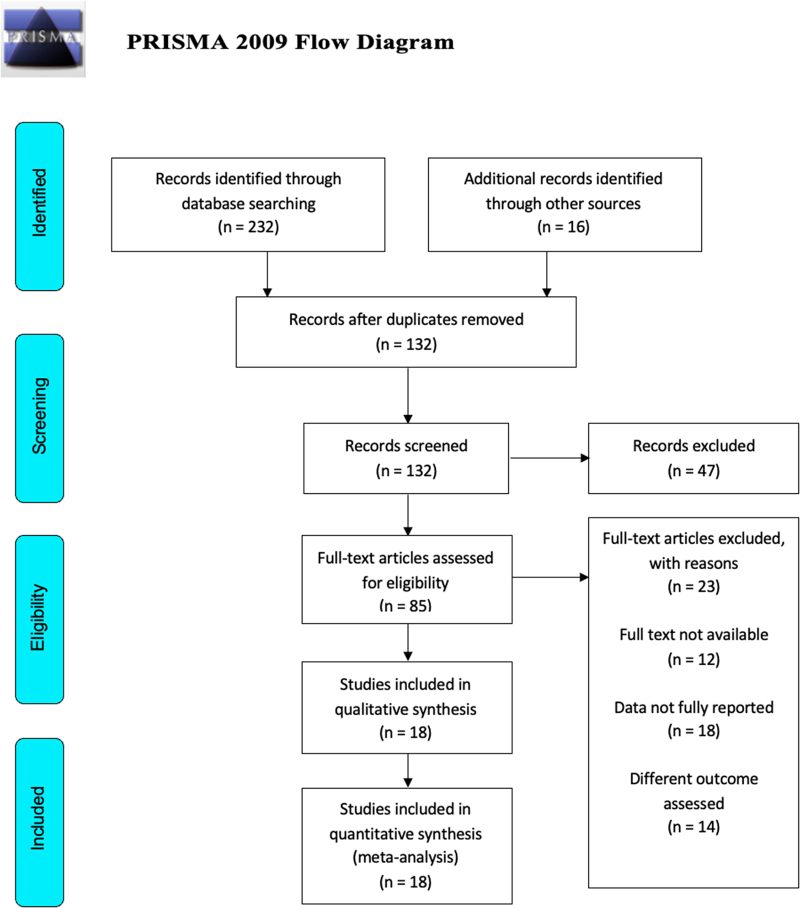
PRISMA 2020 flow diagram for new systematic reviews which included searches of databases and registers only. PRISMA, Preferred Reporting Items for Systematic Review and Meta-Analyses.

The PRISMA flow chart below summarizes the search and study selection process (Fig. 1). The initial search yielded a total of 248 results. After screening and removal of duplicates, 132 articles were assessed for eligibility, and 85 were selected for full-text review. With further exclusion, one retrospective cohort study and 18 RCTs, with a total of 1706 participants, were shortlisted for data extraction.^[17–35]^ A total of 1706 participants were included in our study, of which 861 were randomized to the music therapy group and 845 were randomized into the control group. Table 1 shows the baseline and study characteristics of the included studies.

**Table 1:** Baseline characteristics of the included studies.

			Participants (*n*)		Mean age (SD)		Males (%)		Marital status				Literacy			
Study name	Year of study	Study design	Experimental	Control	Experimental	Control	Experimental	Control	Experimental (*n*)		Control (*n*)		Experimental (*n*)		Control (*n*)	
									Married	Other	Married	Other	Literate	Illiterate	Literate	Illiterate
Twiss et al	2006	RCT	42	44	72.6 (2.1)	75.1 (3.4)	33	33	NA	NA	NA	Na	NA	NA	NA	NA
Hamel et al	2001	RCT	51	50	57 (9)	56 (6)	66	58	NA	NA	Na	NA	NA	NA	NA	NA
Cadigan et al	2001	RCT	75	65	62 (11.4)	62.5 (14)	75	68	48	15	44	20	NA	NA	NA	NA
Taylor-Pilliae et al	2002	RCT	15	15	56.9 (10.3)	65 (6.9)	83	73	27	3	14	1	30	0	15	0
Bally et al	2003	RCT	56	51	59 (11)	58 (11)	59	55	NA	NA	NA	NA	NA	NA	NA	NA
Argstatter et al	2006	RCT	28	27	65.8 (8.4)	67.5 (14)	56	57	NA	NA	NA	NA	NA	NA	NA	NA
Nilsson et al	2012	RCT	34	34	67 (10)	65 (9.9)	-	-	NA	NA	NA	NA	NA	NA	NA	NA
Nilsson et al	2009	RCT	28	30	64 (11.5)	69 (7.5)	-	-	NA	NA	NA	NA	NA	NA	NA	NA
Dogan et al	2012	RCT	100	100	NR	NR	71	69	99	1	91	4	53	47	35	65
Forooghy et al	2015	RCT	32	32	60 (9.53)	56.78 (8.47)	69	59.4	30	2	32	0	24	8	29	3
Rejeh et al	2016	RCT	65	65	61.52 (12.65)	61.84 (11.52)	42.2	57.8	43	22	44	21	45	20	48	17
Amiri et al	2017	RCT	45	45	58.61 (9.55)	57.71 (9.88)	60	64.4	39	6	34	2	NA	NA	NA	NA
Barnason et al	1995	RCT	33	34	NR	NR	68	68	NA	NA	NA	NA	62	0	34	0
Buffum et al	2006	RCT	89	81	67.01 (10.29)	66.65 (9.61)	98	98	NA	NA	NA	NA	89	0	81	0
Chan et al	2007	RCT	31	35	NR	NR	80.6	65.7	29	2	32	3	31	0	34	1
Dai et al	2020	Retrospective Study	33	33	53.4 (12.6)	55.8 (10.9)	54	57	30	3	31	2	33	0	33	0
Dong et al	2023	RCT	43	43	57.6 (12.7)	54.8 (11.4)	58.8	60.5	33	10	34	9	43	0	43	0
Heidari et al	2015	RCT	30	30	56.33 (13.52)	60.91 (8.66)	50	60	27	3	26	4	18	12	21	9
Çelik et al	2022	RCT	31	31	58.58 ± 8.14	56.81 ± 9.54	NA	NA	NA	NA	NA	NA	NA	NA	NA	NA

NR, not reported; RCT, randomized controlled trials.

### Outcomes

#### Anxiety levels using the STAI

**Figure 2.**
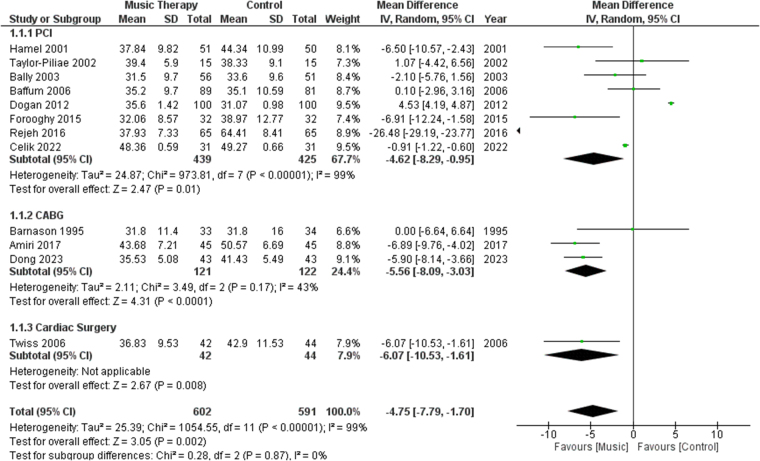
Forest plots comparing anxiety levels, measured by STAI, of patients who received music therapy versus the control group. STAI, State-Trait Anxiety Inventory.

A total of 12 studies measured anxiety levels using the STAI. We performed subgroup analysis based on procedures involved in the study, such as PCI, CABG, or cardiac surgery. Analysis showed a significant difference between the music therapy group and the control group in terms of lowering anxiety levels [MD: −4.75, 95% CI: (−7.79, −1.70), *P* = 0.002, *I*^2^ = 99%] (Fig. 2).

#### Anxiety using the NRS-A

**Figure 3.**
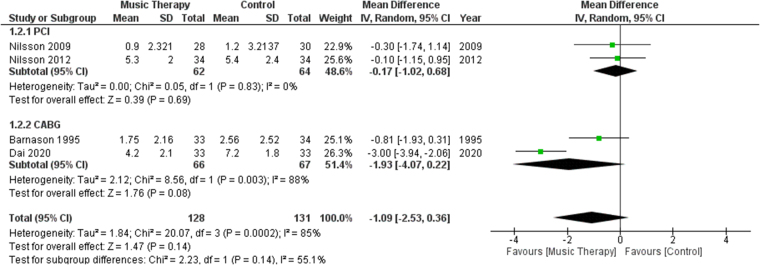
Forest plots comparing anxiety levels, measured by NRS-A, of patients who received music therapy versus the control group. NRS-A, Numerical Anxiety Rating Scale.

Four studies reported anxiety using the NRS-A. A subgroup analysis was performed comparing studies that involved PCI versus those that involved CABG. No significant difference was yielded in terms of anxiety between the music therapy group and the control group [MD: −1.09, 95% CI: (−2.53, 0.36), *P* = 0.14, *I*^2^ = 85%] (Fig. 3).

#### Heart rate

**Figure 4.**
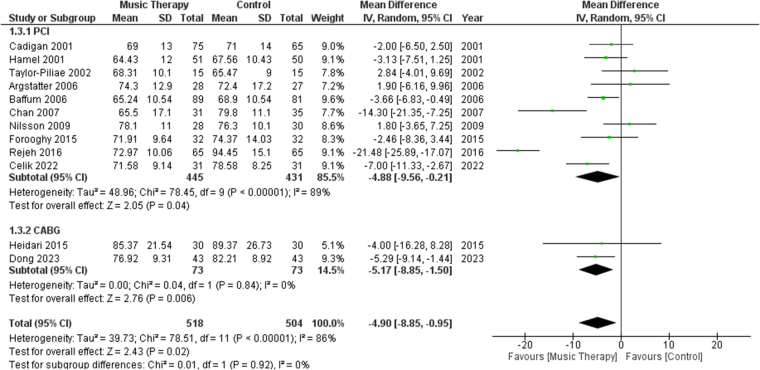
Forest plots comparing heart rates of patients who received music therapy versus control group.

A total of 12 studies reported HR as an outcome. Subgroup analysis was performed between studies performing PCI or CABG. There was a significant difference in terms of HR between the music therapy group and the control group [MD: −4.90, 95% CI (−8.85, −0.95), *P* = 0.02, *I*^2^ = 86%] (Fig. 4). When sensitivity analysis was performed, heterogeneity for HR fell to 49% from 86% after excluding data from Rejeh^[18]^ and Nilsson^[20]^ (Fig. S3, http://links.lww.com/MS9/A783].

#### SBP

**Figure 5.**
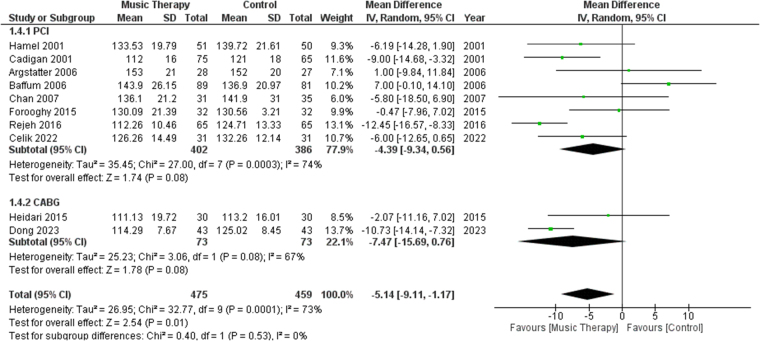
Forest plots comparing SBP of patients who received music therapy versus the control group. SBP, systolic blood pressures

Ten studies reported SBP in their patients after the procedure. Subgroup analysis was performed between studies involving PCI or CABG. There was a significant difference in terms of SBP between the music therapy group and the control group [MD: −5.14, 95% CI (−9.11, −1.17), *P* = 0.01, *I*^2^ = 73%] (Fig. 5). When sensitivity analysis was performed, heterogeneity for the outcome of SBP fell to 37% from 73% after excluding data from Rejeh^[18]^ and Baffum^[31]^ (Fig. S4, http://links.lww.com/MS9/A783). The heterogeneity was further reduced to 0% after removing data from Dong^[25]^.

#### DBP

**Figure 6.**
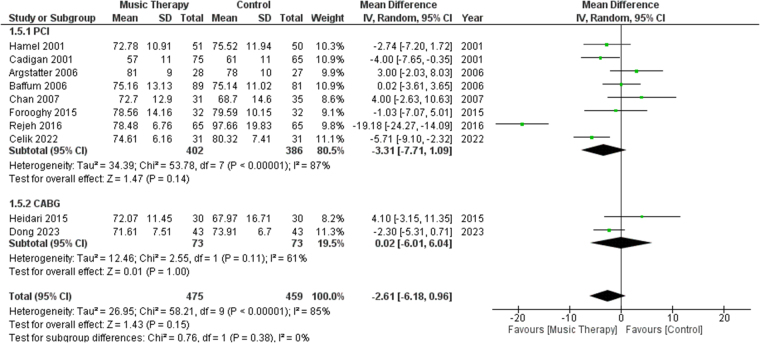
Forest plots comparing DBP of patients who received music therapy versus the control group. DBP, diastolic blood pressure.

A total of 10 studies reported post-procedure DBP. We performed a subgroup analysis of the studies involving PCI or CABG. No significant difference was found in DBP between the music therapy group and the control group [MD: −2.61, 95% CI (−6.18, 0.96), *P* = 0.15, I^2^ = 85%] (Fig. 6). When sensitivity analysis was performed, heterogeneity for the outcome of DBP fell to 46% from 85% after excluding data from Rejeh^[18]^ and Argstatter^[34]^ (Fig. S5, http://links.lww.com/MS9/A783).

#### Respiratory rates

**Figure 7.**
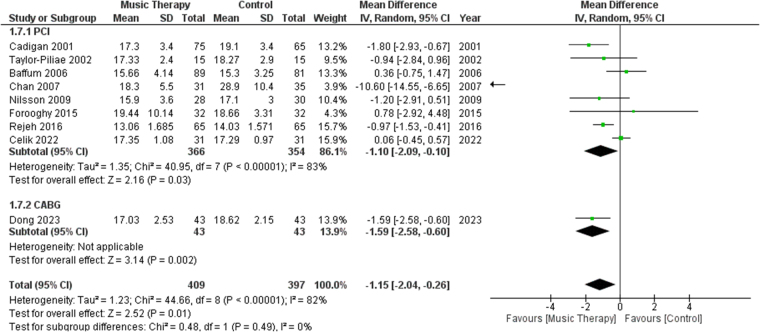
Forest plots comparing respiratory rates of patients who received music therapy versus the control group.

A total of 9 studies reported post-procedure RRs of their patients. Subgroup analysis was done between studies involving PCI and CABG. A significant difference was found in terms of RRs between the music therapy group and the control group [MD: −1.15, 95% CI (−2.04, −0.26), *P* = 0.01, *I*^2^ = 82%] (Fig. 7). When sensitivity analysis was performed, heterogeneity for the outcome of RR fell to 40% from 82% after excluding data from Chan^[28]^ and Çelik^[29]^ (Fig. S6, http://links.lww.com/MS9/A783).

#### MAP

**Figure 8.**
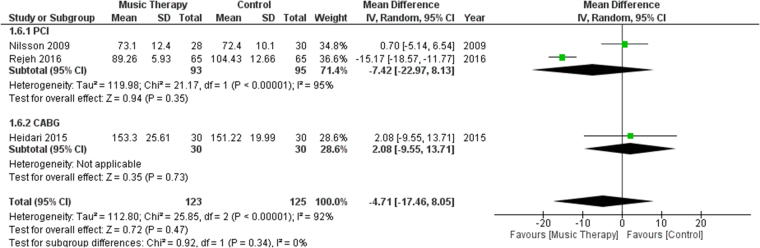
Forest plots comparing MAP of patients who received music therapy versus the control group. MAP, mean arterial pressure.

Three studies reported the MAP of their participants after the procedure. Subgroup analysis was done between studies performing PCI or CABG on their patients. No significant difference was found in terms of MAP between the music therapy group and the control group [MD: −4.71, 95% CI (−17.46, 8.05), *P* = 0.47, *I*^2^ = 92%] (Fig. 8).

#### SaO_2_

**Figure 9.**
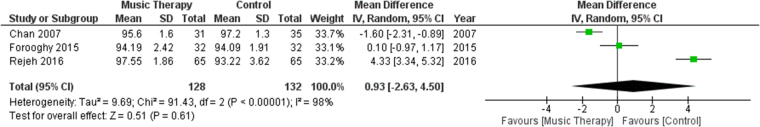
Forest plots comparing SaO_2_ of patients who received music therapy versus the control group. SaO_2_, arterial oxygenation.

Three studies performing PCI reported post-procedure SaO_2_ of the participants, and no significant difference was yielded between the music therapy group and the control group [MD: 0.93, 95% CI (−2.63, 4.50), *P* = 0.61, *I*^2^ = 98%] (Fig. 9).

## Discussion

This meta-analysis assessing the effectiveness of music therapy on anxiety levels among patients undergoing cardiac procedures shows several key findings. First, anxiety levels, as measured by STAI, were significantly reduced in the music therapy group, but no reduction was found when measured by NRS-A. Secondly, patients receiving music therapy showed significant reductions in HR s. Lastly, SBP, but not DBP, showed a significant reduction among the music therapy groups. These results are important because they can help guide clinical treatment and improve the quality of care among patients undergoing cardiac procedures.

In a previous meta-analysis by Su et al., a statistically significant difference was seen in anxiety levels between the music therapy group and the control group among patients undergoing PCI^[36]^. Secondary outcomes like BP and HR showed no significant difference. Compared to this study, we included patients undergoing PCI and CABG, and by having nine new studies, we comprehensively analyzed the current literature to provide a robust comparison. In this updated meta-analysis, which incorporates nine recent articles and considers patients undergoing PCI and CABG in comparison to Su et al. (only PCI), we reanalyzed the data. We found a significant decrease difference in anxiety levels with the use of music therapy when measured using STAI. However, certain secondary outcomes such as HR and BP also showed a significant difference in contrast to the previous study.

Our review demonstrates a significant decrease in anxiety among patients undergoing Cardiovascular procedures (PCI or CABG) when assessed and measured through STAI. However, when assessing anxiety by NRS-A, a non-significant difference was observed. This discrepancy could be explained due to the difference in sensitivity of the two scales. While STAI is a multidimensional tool that provides a comprehensive report of a patient’s psychological state, NRS-A is a numerical scale and may miss subtle changes in anxiety levels. Numerical scales such as NRS-A also rely on self-reporting, which may be more subjective than STAI and introduce bias^[37,38]^.

Music therapy also proved to be an effective intervention for secondary outcomes like HR, SBP, and RR, all showing a significant difference compared to the control group. This is in accordance with other studies like that of Darki et al^[39]^. Their review, like ours, also performed stratification based on age, and both studies still reported a significant difference in HR with music therapy; this indicates the validity of the results^[39]^. In contrast, the study of Heidari et al. showed no significant difference in HR and BP with music therapy^[22]^. These differences could be explained by the different ways music therapy was used. While patients in the latter study were asked to listen to nature calls like sea and bird sounds, Baffum^[31]^ and Celick’s^[29]^ studies allowed the patients to play the music of their own choice. Music played at different tempos could have different effects on HR and BP. Slow-paced music is known to decrease SBP and reduce HR, while fast-paced music may increase HR^[39,40]^.

Our meta-analysis revealed significant heterogeneity (*I*^2^ = 99%) in anxiety reduction measured by the STAI. This variability may stem from differences in patient populations, including baseline anxiety levels, cultural influences, and prior exposure to music therapy. Additionally, the timing and setting of interventions varied, with some studies applying music therapy pre-procedure, while others implemented it intra- or post-procedure. Variability in music delivery (headphones versus ambient music), genre, and session duration further contributed to inconsistencies. Music therapy interventions included classical, instrumental, nature sounds, and patient-preferred selections, with session durations ranging from 10 to 45 minutes. Some studies allowed patients to choose their music, while others used pre-selected tracks. Slow-paced music (60–80 beats per minute) was generally more effective in reducing anxiety. These differences underscore the need for standardized protocols in future studies to optimize music therapy interventions for cardiac patients and improve comparability across studies.

The findings of this review can serve as a helpful guide in clinical practice and improve the quality of care while simultaneously decreasing adverse outcomes in patients undergoing PCI or CABG. The possibility of music therapy as an adjuvant intervention in cardiovascular operations is suggested by its effectiveness in reducing anxiety and secondary physiological markers, including BP and HR. Optimizing therapeutic advantages may be achieved by customizing music treatments according to patient preferences, including loudness, pace, and style of music. Standardized protocols should be the goal of future research, given the variation seen in music therapy sessions and their impact on physiological markers and anxiety. Research comparing various musical genres, tempos, and their effects on therapeutic outcomes could provide more specialized and efficient interventions.

The current review has certain limitations. Some outcomes, like anxiety, had a very high heterogeneity, which prompted us to perform a subgroup and sensitivity analysis to better understand why the risk of bias was so high. Despite these efforts, heterogeneity for anxiety remained high. This could be due to diverse interventions used by different studies, such as music type, tempo, volume, and duration. Moreover, people’s taste in music varies widely depending on their experiences, socio-economic background, and diverse cultures. The subjective preferences and various methodologies used by different studies might contribute to inconsistent results.

## Conclusion

In conclusion, this study underscores the positive impact of music therapy on reducing anxiety levels in patients undergoing cardiac procedures, particularly PCI and CABG. The findings reveal a significant decrease in anxiety, HR, and SBP, supporting the potential of music therapy as a non-pharmacological intervention to enhance patient well-being during cardiovascular interventions. Future research should focus on standardized protocols and explore the nuanced effects of different musical elements on patient responses.

## Data Availability

All data included in the manuscript.

## Research registration unique identifying number (UIN)

Unique identifying number (UIN): CRD42024587231, https://www.crd.york.ac.uk/prospero/display_record.php?ID=CRD42024587231.
